# COVID‐19 Pandemic Imperils Weather Forecast

**DOI:** 10.1029/2020GL088613

**Published:** 2020-08-03

**Authors:** Ying Chen

**Affiliations:** ^1^ Lancaster Environment Centre Lancaster University Lancaster UK

**Keywords:** COVID‐19 pandemic, weather forecast, aircraft, assimilation, accuracy

## Abstract

Weather forecasts play essential parts in economic activity. Assimilation of meteorological observations from aircraft improves forecasts greatly. However, global lockdown during the COVID‐19 pandemic (March to May 2020) has eliminated 50‐75% aircraft observations and imperiled weather forecasting. Here, we verify global forecasts against reanalysis to quantify the impact of the pandemic. We find a large deterioration in forecasts of surface meteorology over regions with busy air flights, such as North America, southeast China, and Australia. Forecasts over remote regions are also substantially worse during March to May 2020 than 2017–2019, and the deterioration increases for longer‐term forecasts. This could handicap early warning of extreme weather and cause additional economic damage on the top of that from the pandemic. The impact over Western Europe is buffered by the high density of conventional observations, suggesting that introduction of new observations in data‐sparse regions would be needed to minimize the impact of global emergencies on weather forecasts.

## Key Points

- The COVID‐19 pandemic eliminated 50–75% of aircraft meteorological observations
- Accuracy of weather forecast reduced significantly especially over southeast China and USA, and error develops as forecast goes longer
- This could handicap early warning of extreme weather; establishing more meteorology stations can buffer the impact of global emergencies

## 1 Introduction

Weather forecasts play an essential part in daily life (Böcker et al., 2013), agriculture (Calanca et al., 2010), and industrial activities (Teisberg et al., 2005) and have great economic value (Zhu et al., 2002). The accuracy of forecasts is largely dependent on the quality of initial conditions used in numerical weather prediction models. The number of meteorological observations has increased steadily over the past decades globally, and their assimilation has greatly improved model initial conditions and forecasts (Kanamitsu, 1989). Aircraft observations from commercial airlines around the world are a critical component of global meteorological observations. Assimilation of aircraft observations exerts the largest improvements in global weather forecasts compared with each individual category of conventional observations (exclude satellite), both for long‐term average and for individual events (Ota et al., 2013; Petersen, 2016).

However, availability of these critical aircraft observations has reduced remarkably since March 2020, resulting from the global lockdown in response to the COVID‐19 pandemic. According to the International Civil Aviation Organization, by the end of March 2020, more than 20 commercial airlines have stopped flights entirely and about 12 airlines stopped all international flights. This eliminates about 50–75% of aircraft observations globally during March to May 2020, according to the World Meteorological Organization (WMO; WMO, 2020), the European Centre for Medium‐Range Weather Forecasts (ECMWF; ECMWF, 2020) and the Aircraft Meteorological DAta Relay program (https://amdar.noaa.gov). Lack of critical aircraft observations could imperil the weather forecast. WMO, ECMWF, and scientists expressed concerns over the impacts to the public regarding the possibility of unreliable weather forecasts (ECMWF, 2020; Viglione, 2020; WMO, 2020). The lack of aircraft data may become worse as the COVID‐19 pandemic develops further and the associated lockdown extends, and this will lead to larger impacts on weather forecasting and impose an additional economic cost on top of that from the pandemic itself. Therefore, a quantifying understanding of the potential impacts of the pandemic on weather forecasting and development of mitigation approaches are critical for protecting current living standards and economic activity.

In this study, we quantify the impact of the COVID‐19 pandemic on weather forecasts by verifying global weather forecast against reanalysis data, which is the best available estimate of the atmospheric state. We also present the difference in impacts over different regions. Based on scientific evidences, we provide suggestions to minimize the impact of global emergencies, such as the COVID‐19 pandemic, on weather forecasting in future.

## 2 Materials and Methods

### 2.1 COVID‐19 Pandemic Impacts on Aircraft Meteorological Observations

Coronavirus disease 2019 (COVID‐19) broke out globally during February 2020 and became a global pandemic in March 2020 (WHO, 2020). Since March, lockdown has been enforced by countries across the world to control the spread and save lives. For example, Italy announced lockdown on 9 March, followed by Spain on 14 March, France on 17 March, Germany on 22 March, UK on 23 March, the United States banned travel from Europe since 14 March^,^ and Australia banned all international visitors since 19 March. These restricting measures have produced a remarkable reduction in meteorological observations from commercial airlines since March 2020 (WMO, 2020).

Aircraft Meteorological DAta Relay program (AMDAR), initiated by WMO, includes more than 3,500 aircraft from ~40 commercial airlines globally and provides more than 680,000 temperature and wind reports per day (Petersen, 2016). About 100 aircraft, mainly over the United States, can also provide moisture observations (Petersen, 2016). These observations are reported every few minutes when aircraft are at cruise levels and every few seconds for profiles during take‐off or landing. As part of WMO protocols (Moninger et al., 2003), these AMDAR observations are quality controlled by National Centers for Environmental Prediction (NCEP). The AMDAR data set has the highest density over North America and Europe, where the airspace is busiest, but these regions are also the centers of the COVID‐19 pandemic in March to May 2020. Due to lockdown during the pandemic, the total number of meteorological profile aircraft reports reduced by more than 50% from about 102,000 per week in February 2020 to about 52,000 in the last week of March 2020, and further reduced to about 26,000 in the last week of April and May 2020 (data source: https://amdar.noaa.gov).

### 2.2 Weather Forecast Data Set and Observation‐Based Data Sets

In order to investigate the impact of the reduction in aircraft observations on global weather forecasts in March to May 2020, the NCEP Global Forecast System (GFS) data set (ds084.1; NCEP, 2015a) is verified against the high‐resolution NCEP Global Data Assimilation System (GDAS) reanalysis data set (ds083.3; NCEP, 2015b) and Global Precipitation Climatology Centre monthly precipitation data set (GPCC; Ziese et al., 2011). The GFS model couples atmosphere, ocean, land/soil, and sea ice modules to produce the weather forecast. There are 64 hybrid sigma‐pressure layers in the atmospheric module, from the ground surface to about 0.27 hPa (Sela, 2009). More details of GFS model are given in the website of NCEP‐GFS (NCEP, 2019). The GDAS data are our best estimate of the atmospheric state; they assimilate the greatest number of meteorological observations from global sources, including aircraft, radiosonde, satellite‐based and ground‐based observations; details given in NCEP (2018). GPCC data provides monthly total precipitation over land surface on a global scale at a resolution of 1.0° × 1.0° (latitude × longitude), based on the surface synoptic observations from WMO *(*Ziese et al., 2011). GDAS and GFS data sets with a horizontal resolution of 0.25° are adopted, and forecast results up to 192 hr are analyzed in this study.

We demonstrate the reduction of forecast accuracy in temperature, relative humidity (RH), wind speed, and pressure with a special focus on temperature, because temperature is widely observed by commercial airlines with high quality and assimilated in the GDAS (Petersen, 2016). In this study, we mainly focus on the surface layer and at 00:00 UTC. These GDAS reanalyses are believed to be the highest‐quality ones, since most surface meteorological observations are still working properly during the COVID‐19 pandemic, and the largest availability of radiosonde observations is at 00:00 UTC around the world (Ingleby et al., 2016). We also discuss the impacts of elimination in aircraft observations during the pandemic on precipitation forecasts by validating GFS forecasts against the observation‐based GPCC data set.

To investigate the impact of the COVID‐19 pandemic on the weather forecast, we compare the forecast accuracy for March to May 2020 (during global lockdown) against the average of March to May 2017–2019. In addition, we conduct the same analysis for February 2020 before global lockdown, in order to demonstrate that this impact on accuracy is associated with the pandemic in March to May 2020 rather than the meteorological characteristics of 2020.

## 3 Results and Discussion

### 3.1 COVID‐19 Pandemic Reduces Accuracy of Weather Forecast

**Figure 1:**
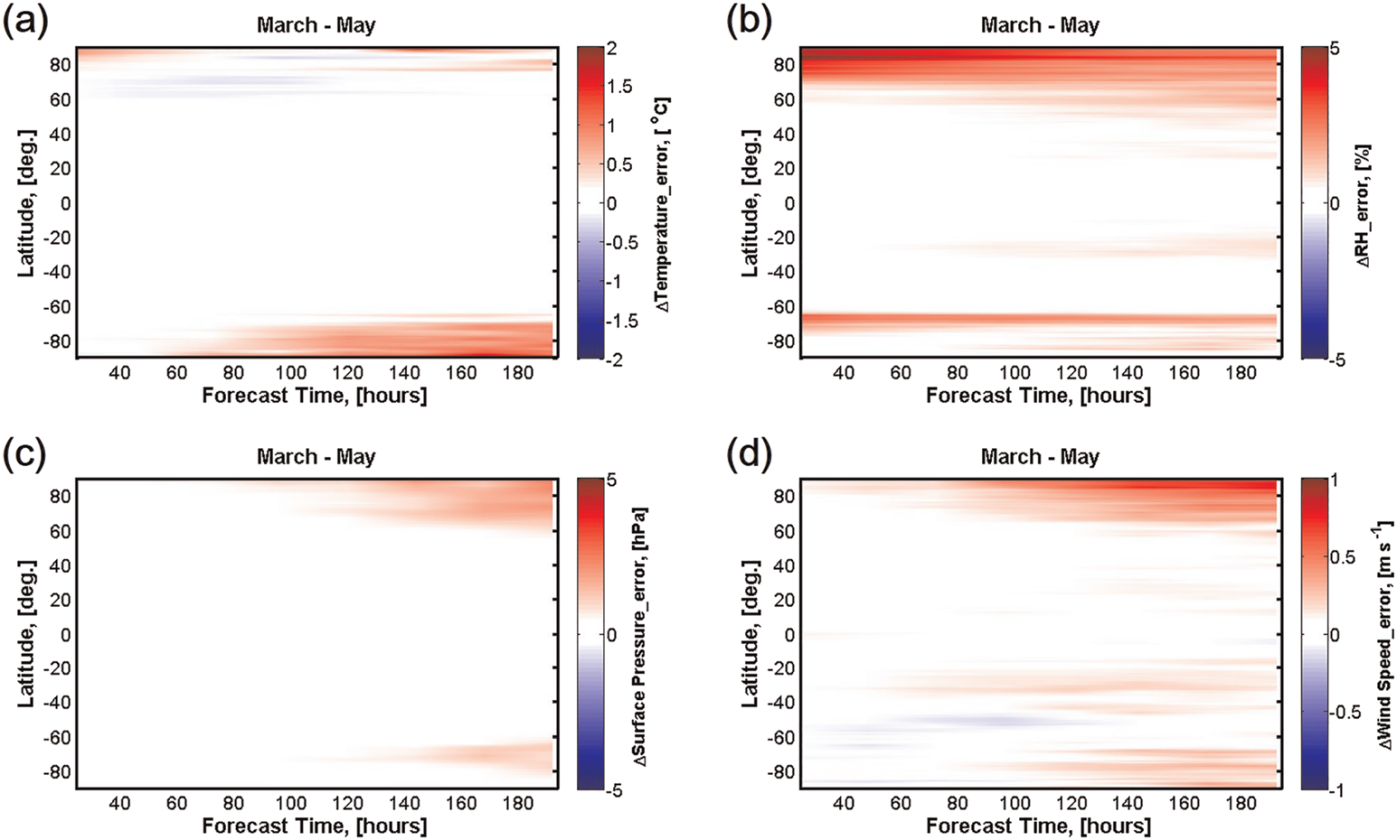
Deviation in absolute error of weather forecasts between 2020 and the average of 2017–2019. Forecasts of 24–192 hr (1–8 days) ahead in the period of March to May, all variables are at 00:00 UTC and in surface layer: (a) temperature; (b) RH; (c) pressure; (d) wind speed. Only deviations with significance higher than 95% confidence level according to *t* test are shown. Red colors indicate worse forecasts in 2020, blue colors indicate better forecasts in 2020.

**Figure 2:**
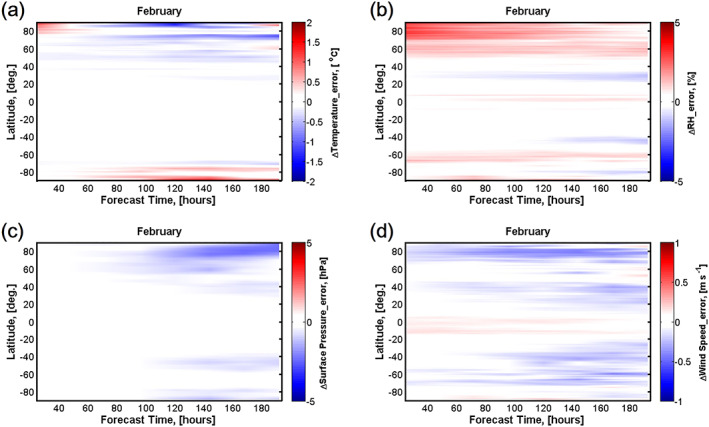
Similar as Figure 1 but forecasts for February. Red colors indicate worse forecasts in 2020, blue colors indicate better forecasts in 2020.

As shown in Figure 1, the accuracy (absolute error) of surface meteorology forecast in March to May 2020 decreases remarkablely (with respect to March to May 2017–2019; red colors indicate worse forecasts, blue colors indicate better forecasts) over north and south polar regions (latitude >70°), throughout the 1–8 day forecasts. Temperature forecast in March to May 2020 shows an extra 0.5–1.0°C bias compared with that in March to May 2017–2019 over south polar regions. The deterioration in temperature forecasts over north polar regions is less than that over south polar regions, by an extra 0–0.5°C bias; however, before the global lockdown in February, the temperature forecast over north polar regions is generally improved by 0.5–1.5°C in 2020 against 2017–2019, with small exceptions in the 24–48 hr forecast (Figure 2a). The surface RH, pressure, and wind speed forecasts in March to May 2020 are also remarkably worse than the forecast in February 2020 (Figures 1b–1d and 2b–2d). The deterioration of the temperature forecast in March to May 2020 develops in the upper layers as the forecast is extended, with large deterioration (~1.0°C) over polar regions from ground up to ~300 hPa in the 168‐hr forecast (details in Figure S1 in the supporting information). This could lead to larger uncertainties in longer forecasts in the descriptions of atmospheric stratification and synoptic scale weather systems, with impacts on medium‐range (3–10 days ahead) to long‐range (15–30 days ahead) forecasts. And the deterioration in global model accurary could worsen predictions for mesoscale and microscale systems using high‐resolution models, whose boundary conditions are constrained by global produces.

No notable deterioration in the surface pressure and wind speed forecasts in March to May 2020 is observed in 24–96 hr forecasts (Figures 1c and 1d), but there is a slight improvement in February 2020 (Figures 2c and 2d). However, the errors develop as the forecasts are extended. In northern polar regions, the 96–192 hr forecasts of surface pressure are worsened by 1–3 hPa in March to May 2020, even though an improvement of 1–4 hPa is seen in the February results (Figure 2c). Similar for wind speed forecast, error in March to May forecasts develops as the forecasts are extended and the accuracy is worsened by up to 0.8 m/s in north polar regions when forecast is more than 100 hr ahead. However, wind forecast of February 2020 shows an improvement by 0.2–0.5 m/s against February 2017–2019, throughout 24–192 hr forcasting period (Figure 2d). Very limited diurnal variation in the deteriorations is observed (Figure S2), indicating that these deteriorations in the forecasts of surface meteorology are consistent throughout a day.

**Figure 3:**
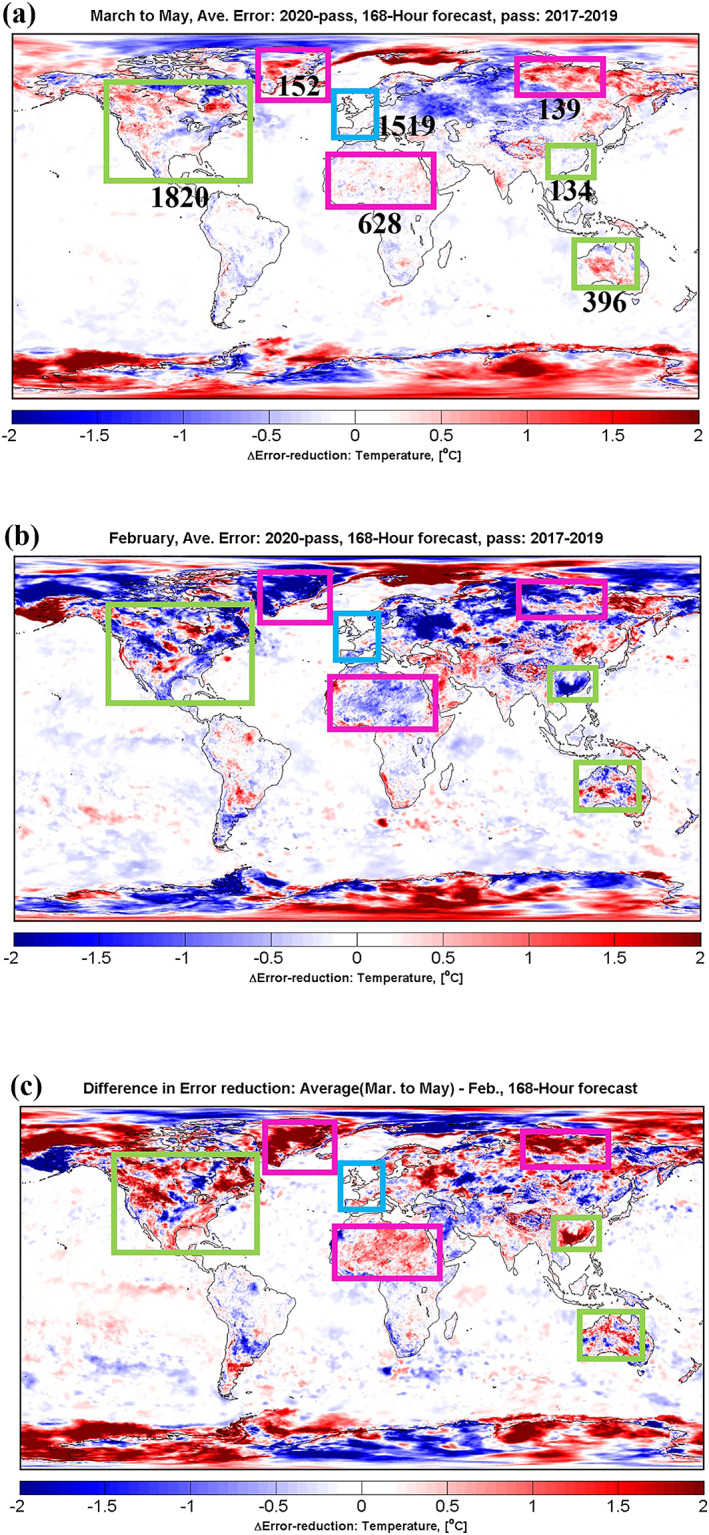
Global map of deviation in absolute error of surface temperature forecasts between 2020 and the average of 2017–2019. The results are for March to May (a) and February (b). The 168‐hr forecasts at 00:00 UTC in surface layer are shown. Only deviations with significance higher than 95% confidence level according to *t* test are shown. The number of meteorological stations in different regions (boxes) are also marked, data sourced from WMO (https://oscar.wmo.int/). Green boxes indicate the regions with busy air flights and large degradation in forecasts, light blue box indicates the region with busy air flights and moderate degradation in forecast, and magenta boxes indicate the remote regions with large degradation in forecasts. The difference between Figures 3a and 3b is shown in Figure 3c. In all panels, red colors indicate worse forecasts, blue colors indicate better forecasts.

The total precipitation forecasts during March to May 2020 are validated against the observation‐based GPCC data set and compared the accuracy in March to May 2020 with March to May in 2017–2019 (Figure S3). No significant deterioration in precipitation forecasts during March to May 2020 is observed, compared with 2017–2019. Although there is some deterioration in a small area of southeast China, the deterioration in precipitation forecasts does not consistently present over a large scale of the regions with busy air flights as the deterioration in temperature forecasts does (Figure 3, see detailed discussion in the next section). This is not surprising, since previous studies show that aircraft observations play a critical role in the forecasts of temperature, humidity, and wind from troposphere to lower‐stratosphere (James & Benjamin, 2017; Ota et al., 2013; Petersen, 2016); while cloud properties from satellites are important for rainfall forecasts (James & Benjamin, 2017) and are not eliminated during the global lockdown.

In summary, better forecasts of surface meteorology are expected in 2020 as indicated by February results, but significant worse forecasts are shown in March to May 2020. This discrepancy strongly suggests that the COVID‐19 pandemic imperils weather forecasting of surface temperature, RH, pressure, and wind speed due to the lack of aircraft observations during the global lockdown. However, precipitation forecasts are not remarkably affected.

### 3.2 Impact in Different Regions

We notice that the degradation of the weather forecast is more substantial in the Northern Hemisphere than in the Southern Hemisphere. This is because there is a much larger number of aircraft observations in this region to constrain the initial conditions of the forecast model. We notice a much larger degradation in the March to May forecast over some regions than others, as shown by the 168‐hr forecast as an example in Figure 3. Remote regions (magenta boxes), such as the Greenland, Siberia, Antartica, and the Sahara Desert, are impacted greatly. This is because assimilation of aircraft observations provides a much larger improvement in forecasts over regions where very limited conventional observations are available (Ota et al., 2013). Regions with busy air flights are also affected greatly, such as North America, southeast China, and Australia (green boxes in Figure 3). The accuracy of the surface temperature forcasts over these regions is reduced or occasionally slightly improved (0–0.5°C) in March to May 2020 (Figure 3a), but we could reasonably expect a larger improvement of 0.5–1.5°C over these regions as seen in the February result (Figure 3b). Therefore, this gap of 0.5–1.5°C improvement between forecasts in March to May and February (calculated as “Figures 3a and 3b,” shown in Figure 3c) could be attributed to the lack of aircraft observations during the COVID‐19 pandemic. This is supported by the reduced availability of aircraft observations as discussed in section 2, where only about 25–50% of aircraft observations were available globally during March to May 2020 compared with February 2020.

As reported in previous studies (Ota et al., 2013; Petersen, 2016) (see also Figure S4), North America, southeast China, and Australia are regions with a large number of aircraft observations under normal conditions. Western Europe (blue box in Figure 3) also has a large amount of aircraft observations, which reduced greatly during the COVID‐19 pandemic with strict lockdown over most European countries. However, nearly no impact on the surface temperature forecasts is observed. This is because there is a dense network of meteorological stations over western Europe compared with other regions, 1,519 stations in the small blue box of Figure 3 (sourced from WMO: https://oscar.wmo.int/), providing a good constraint on the initial conditions of forecast model and hence a reliable weather forecast. Additional aircraft observations make limited improvement over regions where observation information is almost “saturated” (Ota et al., 2013), such as western Europe. Therefore, the high density of conventional meteorological observations buffers the impact of the COVID‐19 pandemic on weather forecasts over western Europe.

## 4 Summary

Weather forecasts play an essential part in daily life, agriculture, and industrial activities, and their accuracy is largely dependent on the amount of meteorological observations assimilated in forecast models. The COVID‐19 pandemic has led to a global lockdown and greatly reduced the number of flights and the associated aircraft observations during March to May 2020. In this study, we verify global weather forecasts in March to May 2020 against high‐resolution global reanalysis data set and an observation‐based global precipitation data set, which are the best estimate of the atmospheric state. To investigate the forecast deterioration during the pandemic, the forecast accuracy during March to May 2020 is further compared with the average accuracy during March to May 2017–2019. We report a significant deterioration in the forecasts of surface temperature, RH, wind speed, and pressure, but no significant deterioration in precipitation forecast is observed. A similar analysis for February 2020 suggests that the forecast accuracy of surface meteorology could have been expected to improve in 2020 compared with 2017–2019, if aircraft observations were carried out as usual.

Forecasts over remote and busy air flight regions are more vulnerable due to the lack of aircraft observations. Over Greenland and Siberia, the accuracy of surface temperature forecasts could be reduced by up to 2°C, and the deterioration in the forecasts of surface wind speed and pressure develops as the forecasts are extended. Forecasts over North America, southeast China, and Australia are also greatly affected by the COVID‐19 pandemic, but the impact over western Europe is compensated to some extent by the high density of meteorological observations stations available.

The lack of aircraft observations may become more severe as the COVID‐19 pandemic develops and the associated lockdown extends. This study warns that further worsening of weather forecasts may be expected and that the error could become larger for longer‐term forecasts. This could handicap early warning of extreme weather and cause additional hardship for daily life in the near future. The results also highlight that establishing more meteorological stations in observation‐sparse regions and report data to WMO can improve the weather forecast and effectively buffer the impact of global emergencies, such as the COVID‐19 pandemic, in future.

## Conflict of Interest

The author declares no competing financial interest.

## Data Availability Statements

The GFS (ds084.1) and GDAS (ds083.3) global data sets are available from National Centers for Environmental Prediction/National Weather Service/NOAA/ (https://rda.ucar.edu/). The GPCC data set is available from this site (https://opendata.dwd.de/climate_environment/GPCC/).

## Supporting information

Supporting Information S1
