# Supplementary material for: COVID‐19 Pandemic Imperils Weather Forecast
Source: Geophys Res Lett. 2020 Aug 3;47(15):e2020GL088613. doi: 10.1029/2020GL088613 (PMC7404364; doi:10.1029/2020GL088613)
Supplement: Supplementary file 1 — Supporting Information S1 [file GRL-47-e2020GL088613-s001.docx]

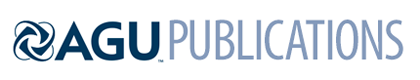


*Geophysical Research Letters*

Supporting Information for

**COVID-19 Pandemic Imperils Weather Forecast**

# Ying Chen^1,*^

*^1^Lancaster Environment Centre, Lancaster University, Lancaster, LA1 4YQ, UK*

**Corresponding to: Ying Chen (y.chen65@lancaster.ac.uk)*

**Contents of this file:**

**Figure S1** – Deviation in absolute error of temperature forecasts;

**Figure S2** – Diurnal variation of deviation in absolute error of meteorological forecasts;

**Figure S3** – Global map of deviation in absolute error of precipitation forecasts;

**Figure S4** – Number of aircraft observations over the world;

| 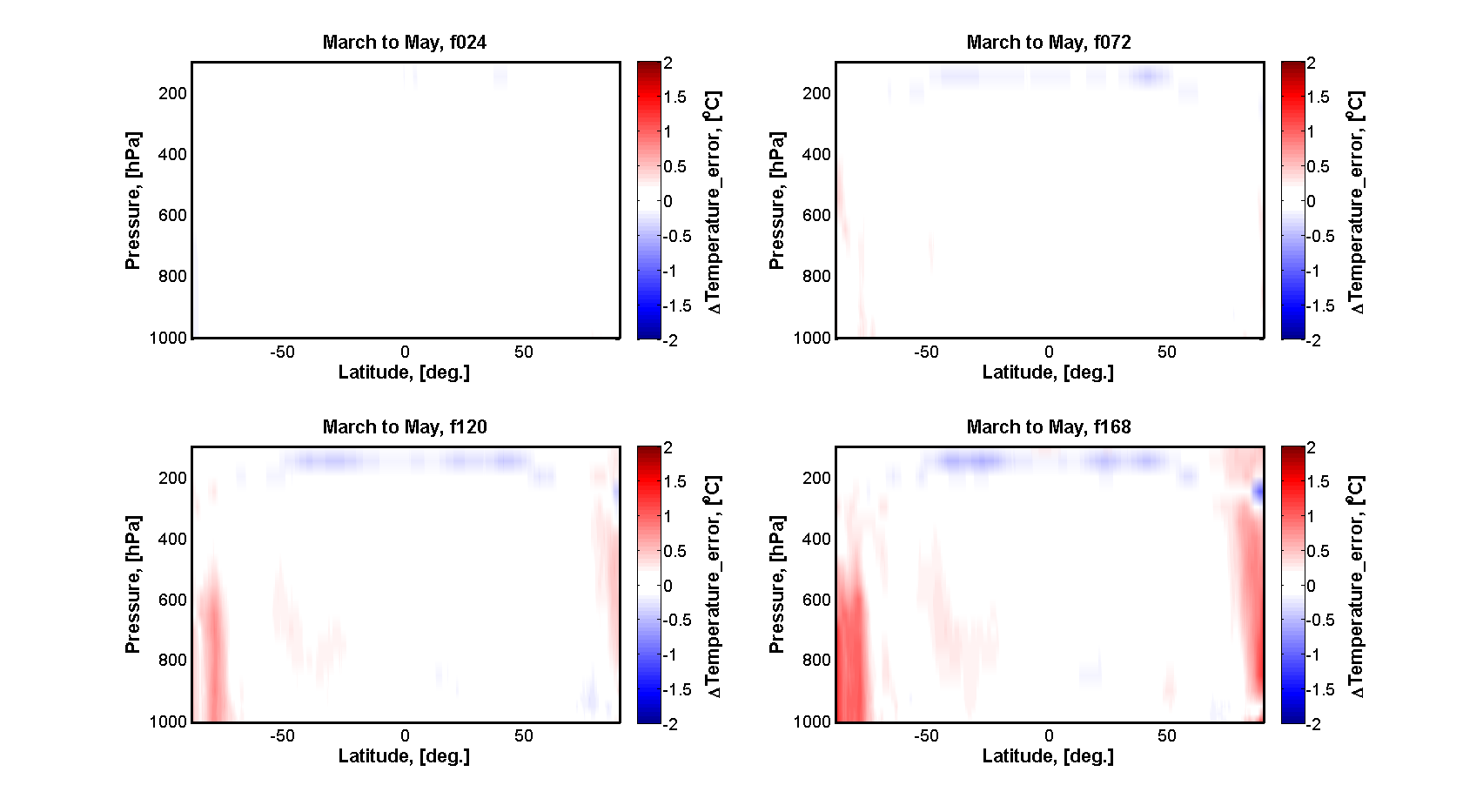  **(a)**  **(b)**  **(c)**  **(d)** |
| --- |
| **Figure S1. Deviation in absolute error of temperature forecasts between 2020 and average of 2017-2019, for the period of March to May.** The results are averaged over longitude. (a) 24-hour forecast; (b) 72-hour forecast; (c) 120-hour forecast; (d) 168-hour forecast. Only deviations with significance higher than 95% confidence level according to t-test are shown. Red colours indicate worse forecast in 2020, blue colours indicate better forecast in 2020. |

| 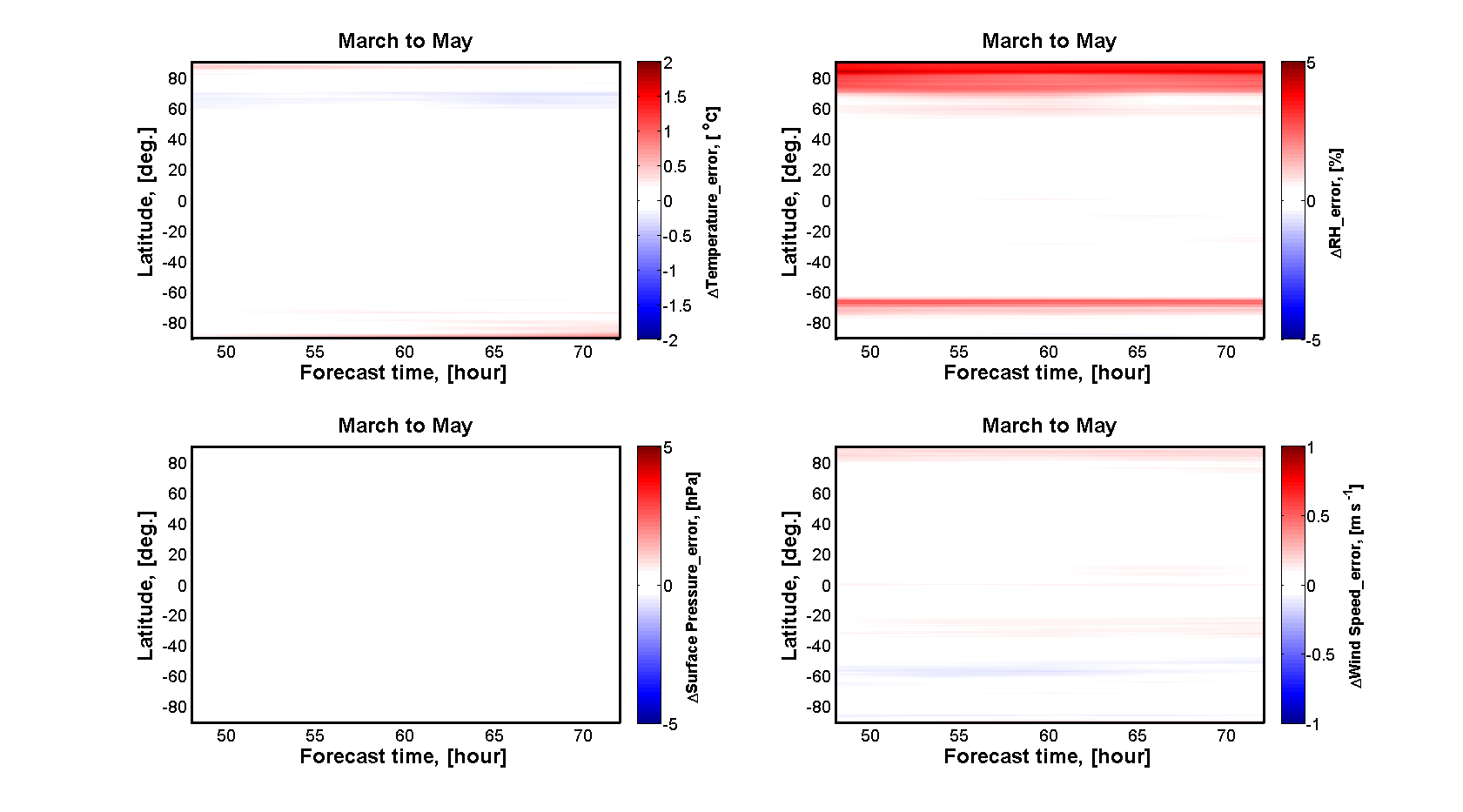  **(a)**  **(b)**  **(c)**  **(d)** |
| --- |
| **Figure S2. Diurnal variation of deviation in absolute error of weather forecasts between 2020 and the average of 2017-2019.** The third-day forecasts (48-72 hours ahead) of surface meteorology in the period of March to May are analysed. The forecasts are initialized at 00:00 UTC. (a) temperature; (b) RH; (c) pressure; (d) wind speed. Only deviations with significance higher than 95% confidence level according to t-test are shown. Red colours indicate worse forecasts in 2020, blue colours indicate better forecasts in 2020. |

| **** |
| --- |
| **Figure S3. Global map of deviation in absolute error of total precipitation forecasts between 2020 and the average of 2017-2019.** The results of 168-hour forecasts during March to May are shown. The results of 24-192 hours (not show here) are similar. Red colours indicate worse forecasts in 2020, blue colours indicate better forecasts in 2020. |

| 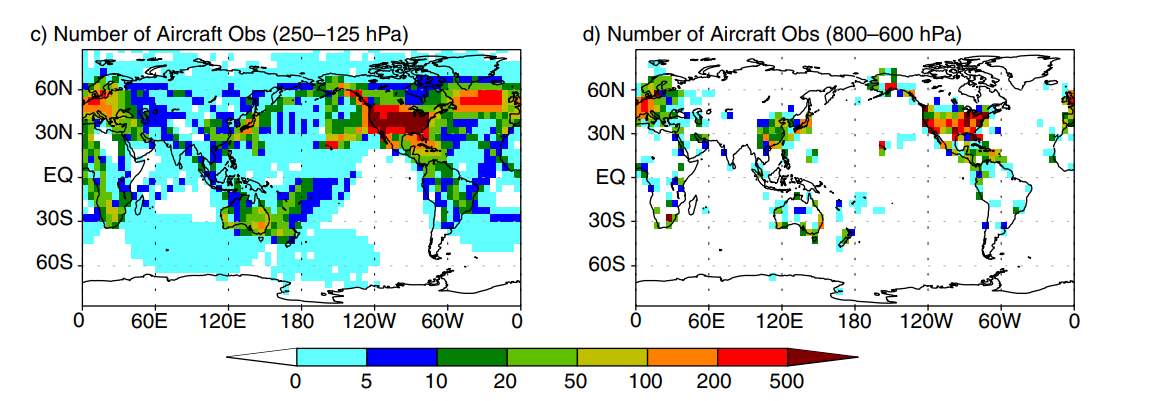  **(b)**  **(a)** |
| --- |
| **Figure S4. Number of aircraft observations over the world.** Source from: [*Ota et al.*, 2013], reuse authorized according to the Creative Commons Attribution-Noncommercial 3.0 Unported License. |

**Supplementary References:**

Ota, Y., J. C. Derber, E. Kalnay, and T. Miyoshi (2013), Ensemble-based observation impact estimates using the NCEP GFS, *Tellus A: Dynamic Meteorology and Oceanography*, *65*(1), 20038.
